# M2-like macrophages in the fibrotic liver protect mice against lethal insults through conferring apoptosis resistance to hepatocytes

**DOI:** 10.1038/s41598-017-11303-z

**Published:** 2017-09-05

**Authors:** Li Bai, Xin Liu, Qingfen Zheng, Ming Kong, Xiaohui Zhang, Richard Hu, Jinli Lou, Feng Ren, Yu Chen, Sujun Zheng, Shuang Liu, Yuan-Ping Han, Zhongping Duan, Stephen J. Pandol

**Affiliations:** 1 Artificial Liver Center, Beijing YouAn Hospital, Capital Medical University, Beijing, 100069 China; 2 Olive View-UCLA Medical Center, Los Angeles, CA 91342 USA; 3 Beijing Institute of Liver Diseases, Beijing, 100069 China; 4 The Center for Growth, Metabolism and Aging, the Key Laboratory for Bio-Resource and Eco-Environment, College of Life Sciences, and the National Key Laboratory of Biotherapy, Sichuan University, Chengdu, 610014 China; 5 Cedars-Sinai Medical Center, and Department of Veterans Affairs, Los Angeles, CA90048 USA

## Abstract

Acute injury in the setting of liver fibrosis is an interesting and still unsettled issue. Most recently, several prominent studies have indicated the favourable effects of liver fibrosis against acute insults. Nevertheless, the underlying mechanisms governing this hepatoprotection remain obscure. In the present study, we hypothesized that macrophages and their M1/M2 activation critically involve in the hepatoprotection conferred by liver fibrosis. Our findings demonstrated that liver fibrosis manifested a beneficial role for host survival and apoptosis resistance. Hepatoprotection in the fibrotic liver was tightly related to innate immune tolerance. Macrophages undertook crucial but divergent roles in homeostasis and fibrosis: depleting macrophages in control mice protected from acute insult; conversely, depleting macrophages in fibrotic liver weakened the hepatoprotection and gave rise to exacerbated liver injury upon insult. The contradictory effects of macrophages can be ascribed, to a great extent, to the heterogeneity in macrophage activation. Macrophages in fibrotic mice exhibited M2-preponderant activation, which was not the case in acutely injured liver. Adoptive transfer of M2-like macrophages conferred control mice conspicuous protection against insult. *In vitro*, M2-polarized macrophages protected hepatocytes against apoptosis. Together, M2-like macrophages in fibrotic liver exert the protective effects against lethal insults through conferring apoptosis resistance to hepatocytes.

## Introduction

Liver fibrosis—characterized by the activation of hepatic stellate cell (HSC), the deposition of interstitial extracellular matrix (ECM), and the distortion of the hepatic vasculature architecture—represents a common outcome of most chronic liver diseases, regardless of aetiology. Advanced fibrosis results in cirrhosis, portal hypertension and liver failure which often require liver transplantation^1–3^. The pathological features of liver fibrosis have been extensively studied and well documented. However, the other side of this “double-edged sword”, i.e., the powerful capacity to repair, has long been overlooked. Most recently, increasing attention has been focused on the homeostatic and beneficial effects of liver fibrosis as an evolutionarily conserved wound healing response^4, 5^. Several outstanding works have suggested that fibrosis confers mice more resistance against subsequent acute liver injury^6–8^. Nevertheless, the cellular and molecular mechanisms governing this hepatoprotection are largely unexplored.

Macrophages are innate immune cells with critical roles in host defence, immune regulation, tissue repair and liver regeneration^9, 10^. In recent years, researchers have devoted much attention to the roles of macrophages in regulating the progression or regression of liver fibrosis^2, 9, 11–13^. The bidirectional roles of liver macrophages in the different phases of fibrosis have been revealed^14^. This dichotomous function is intimately associated with the great plasticity and remarkable heterogeneity of macrophages. Macrophages adapt their phenotypes in response to various microenvironmental signals, and exhibit different characteristic markers, gene expression profiles and functions^15–18^. They are broadly delineated into two categories, namely, M1 and M2 macrophages. M1 macrophages are activated by pathogens or toxins (such as LPS), and secrete pro-inflammatory mediators including tumour necrosis factor (TNF)-α, interleukin (IL)-1β, IL-6, inducible NO synthase (iNOS) and matrix metalloproteinases (MMPs), which induces inflammation and liver damage. On the contrary, M2 macrophages are activated by IL-4/IL-13, and release anti-inflammatory or pro-resolving mediators, such as arginase (ARG)-1, mannose receptor (CD206), IL-10 and transforming growth factor (TGF)-β, which mediates wound repair, tissue remodeling and fibrosis^15–18^.

In the present study, we integrated macrophages and their M1/M2 activation into the development of injury resistance in the context of liver fibrosis, and hypothesized that M2-like macrophage is a key contributor to hepatoprotection in this setting. To address this hypothesis, we first verified the hepatoprotective effects of liver fibrosis against various lethal insults, which was accompanied by the innate immune tolerance. Second, we demonstrated the crucial but divergent roles of macrophages in homeostasis and fibrosis through loss-of-function experiments (depletion of macrophages), and ascribed the dichotomous functions of macrophages to their phenotypic heterogeneity (M2-like activation in the fibrotic liver). Then, we confirmed the protective effects of M2-like macrophages against acute challenge through gain-of-function experiments (adoptive transfer of M2-like macrophages). Finally, we dissected the underlying mechanisms of the hepatoprotection conferred by M2-like macrophages, and showed that M2-like macrophages protected hepatocytes against apoptosis *in vitro*. Our findings provide a novel and rational interpretation for injury resistance in the setting of hepatic fibrosis. This work will shed light on the clinical settings associated with acute-on-chronic liver failure (ACLF).

## Results

### Fibrosis protects mice from a variety of acute lethal insults

**Figure 1:**
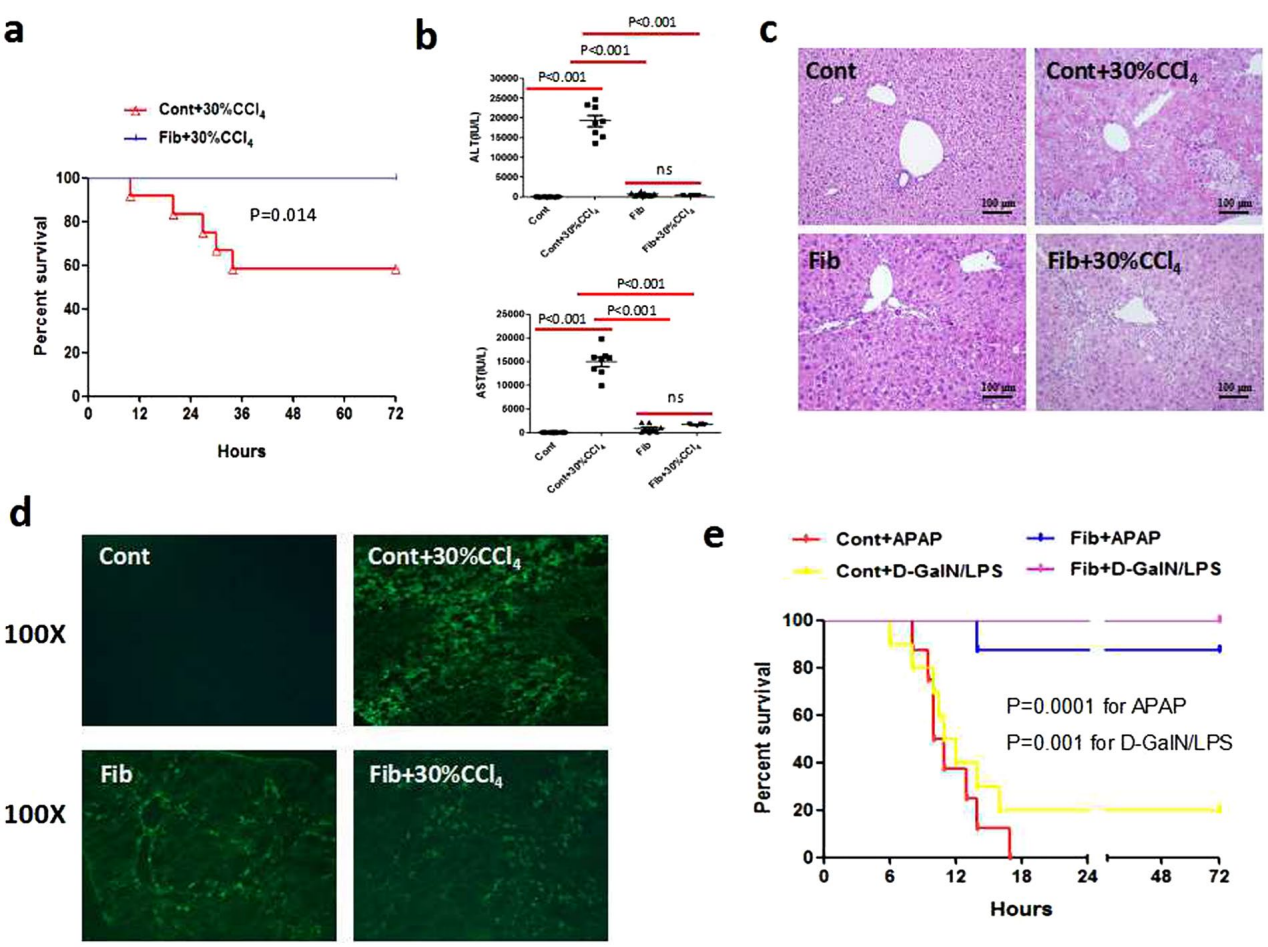
Fibrosis protects mice against various lethal hepatic insults. Control and fibrotic (CCl_4_, 6 weeks) Balb/c mice were challenged with CCl_4_ (3 μl/g), D-GalN (1 mg/g)/LPS (50 ng/g), or APAP (1 mg/g) through intraperitoneal injection. (**a**) The survival rate of control and fibrotic mice upon CCl_4_ challenge (n = 12). (**b**–**d**) The hepatic damage in control and fibrotic mice with or without acute insult was assessed by serum ALT and AST levels, H&E staining and apoptosis detection by TUNEL staining (n = 8). Data were expressed as mean ± SEM. (**e**) The survival rate of control and fibrotic mice subjected to APAP (n = 8) or D-GalN/LPS (n = 10) treatment.

First, we aimed to confirm the beneficial role of liver fibrosis for host survival against hepatic insults. Survival analysis showed that fibrotic mice were fully tolerant to subsequent carbon tetrachloride (CCl_4_) injection (all fibrotic mice survived), whereas only 60% of control mice survived the same dose of CCl_4_ (P = 0.014) (Fig. 1a). Acute insult triggered high levels of alanine aminotransferase (ALT) and aspartate transaminase (AST) in control mice (Fig. 1b). Nevertheless, the fibrotic mice manifested extremely strong tolerance to this insult, as shown by substantially reduced ALT levels (Fig. 1b) and improved hepatic histology (Fig. 1c). Similarly, acute challenge triggered massive apoptosis of liver cells in control mice as evaluated by TUNEL staining, however, cell apoptosis was largely attenuated in fibrotic mice upon insult (Fig. 1d).

We further tested the tolerance of CCl_4_-induced fibrotic mice to additional toxic agents that can induce fulminant liver injury. The majority of control mice (80%, 8 of 10) were sacrificed within 72 h of acute challenge with D-galactosamine/ lipopolysaccharide (D-GalN/LPS). Conversely, fibrotic mice exhibited excellent tolerance to D-GalN/LPS, and no deaths (0%) occurred after acute insult (Fig. 1e). Acetaminophen (APAP) overdose contributes to approximately 50% of cases of acute liver failure (ALF) in Western countries^19^. Here, a single injection of APAP (1 mg/g) led to the death of all control mice (100%, 8 of 8), mostly within 20 h, whereas the majority of fibrotic mice (87.5%, 7 of 8) survived the lethal injection (P = 0.0001) (Fig. 1e). Collectively, liver fibrosis exhibits beneficial effects for host survival and apoptosis resistance.

### Hepatoprotection in the setting of hepatic fibrosis is tightly associated with innate immune tolerance

**Figure 2:**
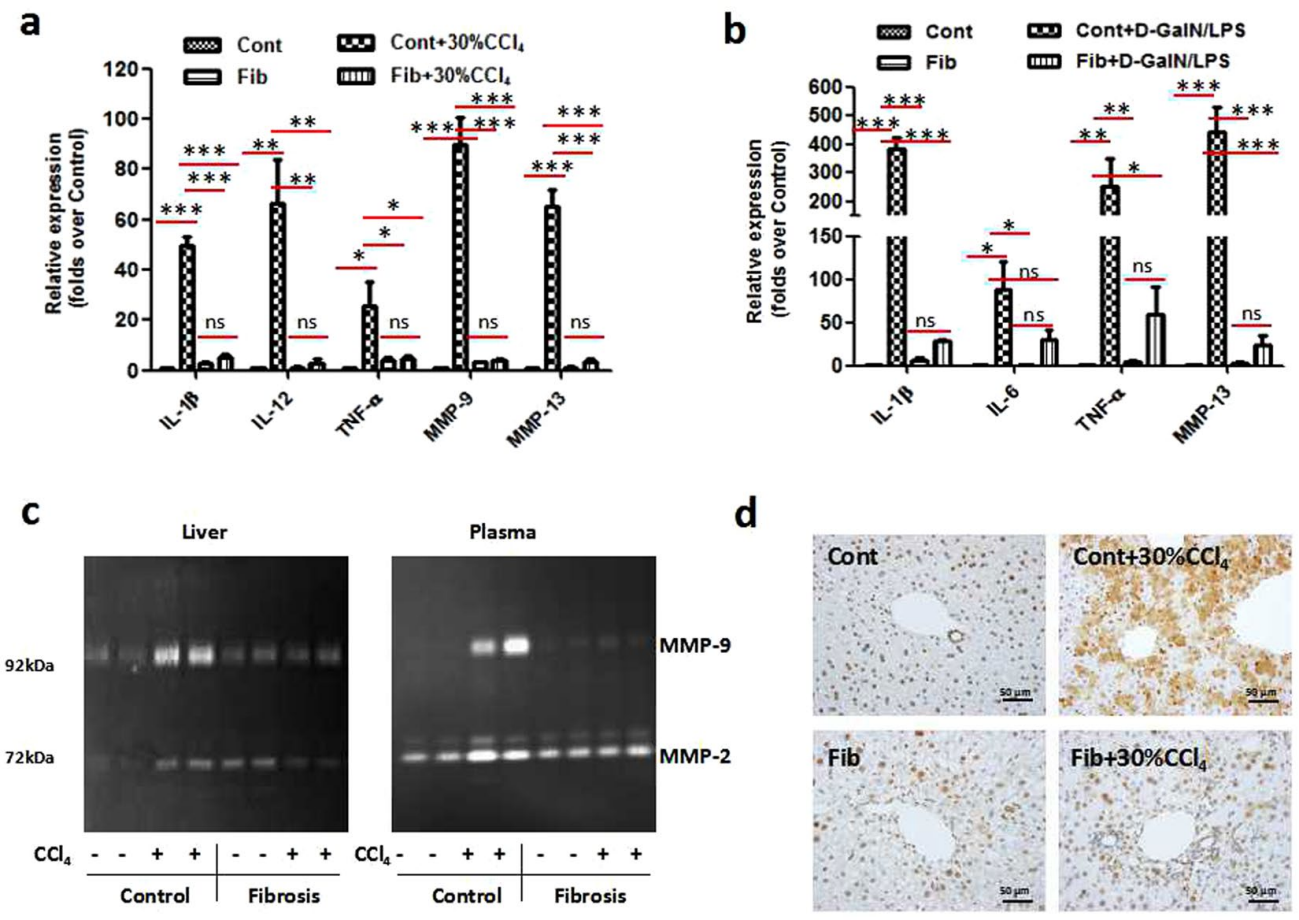
Injury resistance is accompanied by the suppression of pro-inflammatory immune responses. Control and fibrotic (CCl_4_, 6 weeks) mice were challenged with lethal doses of CCl_4_ (**a**,**c**,**d**) or D-GalN/LPS (**b**) (details in *Methods and Materials*). (**a** and **b**) mRNA levels of injury mediators, including IL-1β, IL-12, TNF-α, IL-6, MMP-9, and MMP-13, were detected by qRT-PCR analysis. Data were expressed as mean ± SEM. *p < 0.05, **p < 0.01, ***p < 0.001. (**c**) The MMP-9 activities in the liver tissues and plasma were detected by zymography. (**d**) The expression and translocation of HMGB1 was analysed by immunohistochemistry.

Inflammation is a hallmark of clinical features in virtually all liver diseases. Innate immune responses exert essential roles in the initiation, progression and resolution of hepatic inflammation^20^. In this regard, we analysed the immunological events underlying hepatoprotection in the setting of fibrosis. Pro-inflammatory mediators and matrix metalloproteinases (MMPs), which have been reported to mediate acute injury and lethality^21^, were detected in control and fibrotic mice with or without acute challenge. At the gene level, injury mediators, including IL-1β, IL-12, MMP-9 and MMP-13, were dramatically increased by more than 50-fold in control mice upon acute CCl_4_ challenge. Conversely, although these mediators were moderately expressed in the fibrotic liver, there was no remarkable increase in transcription in response to acute insult (Fig. 2a). We then tested the tolerance of fibrotic mice to an alternative hepatic toxin, D-GalN/LPS. In sharp contrast to the steep increase in hepatic expression of IL-1β, IL-6, TNF-α and MMP-13 in the context of fulminant liver injury induced by D-GalN/LPS, the fibrotic mice were highly resistant to this attack, showing significantly less induction of inflammatory cytokines and MMP (Fig. 2b). Unlike MMP-2, which is constitutively expressed, MMP-9 protein was strongly induced in response to acute injury and released into the plasma, as measured by its gelatinolytic activity (Fig. 2c, Supplementary Fig. 2). Conversely, both the hepatic and plasma MMP-9 activities in the fibrotic mice were refractory to the second insult, which mirrored well with the mRNA levels of MMP-9. High-mobility group box-1 (HMGB1), a potent and classic pro-inflammatory mediator in acute liver injury^22–24^, was also suppressed in the fibrotic liver, even under acute challenge (Fig. 2d). Thus, hepatoprotection in the setting of liver fibrosis is accompanied by the suppression of pro-inflammatory immune responses.

### Macrophages critically involve in the injury resistance and immune tolerance mediated by liver fibrosis

**Figure 3:**
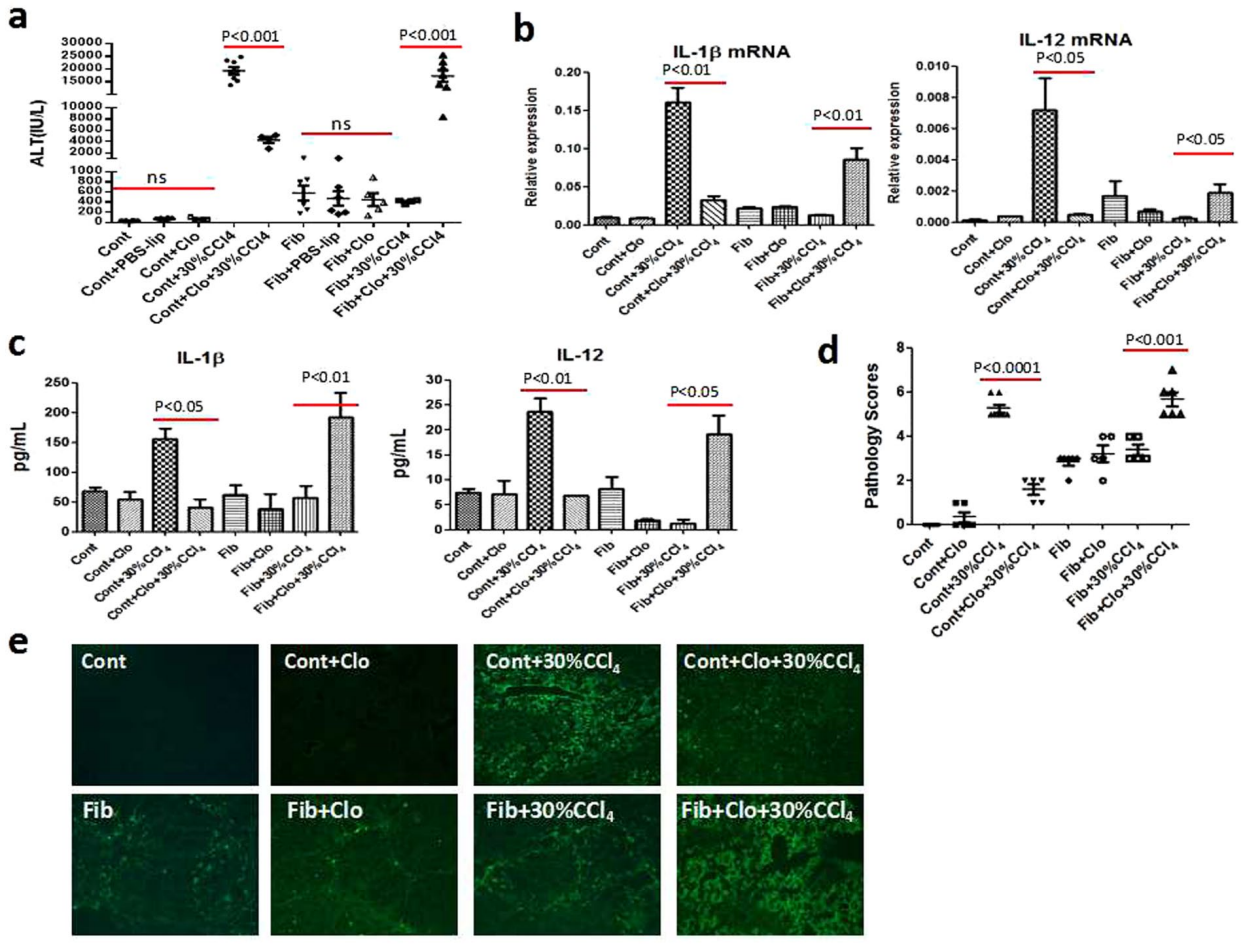
Macrophages exert dichotomous roles in cases of homeostasis and hepatic fibrosis. Macrophages in the control and fibrotic mice were depleted with liposome-clodronate (Clo) for 48 hours, and the resultant mice were then given a lethal dose of CCl_4_ by intraperitoneal injection. Sera and liver tissues were harvested 24 hours after toxin challenge. (**a**) Hepatic injury was assessed by serum ALT levels. (**b** and **c**) The mRNA and protein expressions of injury markers were detected by qRT-PCR and Luminex Assay, respectively. (**d**) Histological severity of liver injury was assessed and scored blindly by experienced pathologists. (**e**) Apoptotic cells were identified using TUNEL staining. Data were expressed as mean ± SEM.

Next, we explored the cellular mechanisms governing the injury resistance and immune tolerance in the setting of liver fibrosis. It is well established that macrophages hold an important position during the progressing and resolving phases of fibrosis^14, 25–27^. Importantly, Kupffer cells (KC), liver resident macrophages, have been reported to exert a protective role for TNF-α-induced apoptosis in fibrotic setting induced by bile duct ligation (BDL)^7^. In light of this, we hypothesized that macrophage is a key contributor to injury resistance and immune tolerance mediated by liver fibrosis. To support our hypothesis, macrophages in control and fibrotic mice were depleted by intravenous injection of liposome-encapsulated clodronate (Clo). Forty-eight hours after Clo treatment, the resultant mice were then subjected to CCl_4_ challenge for another 24 hours. As expected, F4/80+cells were reduced substantially in the fibrotic liver subjected to Clo treatment, indicating that the majority of macrophages had been depleted (Supplementary Fig. 3a). Interestingly, we found that macrophage-depleted control mice manifested remarkably reduced ALT levels upon challenge, as compared to acutely injured mice. Oppositely, acute insult triggered the obvious increase of ALT levels in macrophage-depleted fibrotic mice, which was strikingly inhibited in the fibrotic mice upon insult (Fig. 3a). This finding supports the divergent roles of macrophages under the microenvironments of homeostasis and fibrosis. In addition, the mRNA levels of pro-inflammatory mediators, including IL-1β, IL-6, IL-12, HMGB1 and TNF-α, were down-regulated or exhibited a decreased trend in macrophage-depleted control mice upon CCl_4_ insult, which was totally different from macrophage-depleted fibrotic mice who manifested significant up-regulation of above-mentioned mediators upon insult (Fig. 3b and Supplementary Fig. 3d). Moreover, the protein concentrations of IL-1β, IL-12 and TNF-α paralleled their mRNA levels (Fig. 3c and Supplementary Fig. 3e). Furthermore, histological assessment (Fig. 3d and Supplementary Fig. 3f) and TUNEL assay (Fig. 3e) corresponded well with ALT levels. The administration of Clo resulted in better-preserved liver architecture and significantly reduced apoptotic cells in control mice upon insult. In contrast, the fibrotic mice subjected to Clo treatment displayed exacerbated hepatic damage and massive apoptosis when exposed to acute challenge. Therefore, macrophages assume the critical but divergent roles in the development of injury resistance and immune tolerance.

### Macrophages in the fibrotic liver exhibit M2-preponderant activation

**Figure 4:**
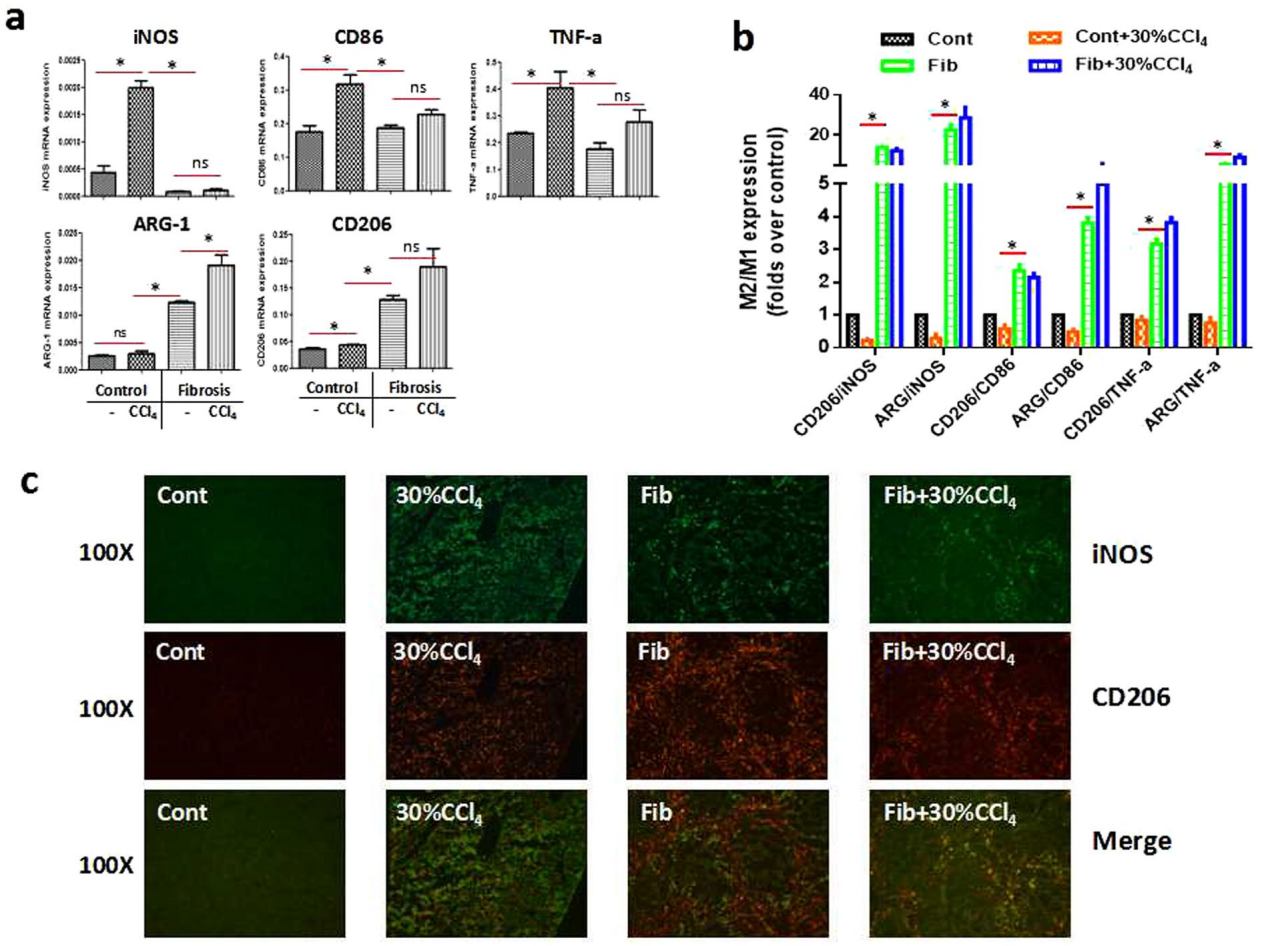
Macrophages in the fibrotic liver exhibit an M2-preponderant signature. Control and fibrotic mice were subjected to a lethal dose of CCl_4_. Twenty-four hours after acute challenge, macrophages were isolated from the livers by pronase/collagenase digestion and differential centrifugation using Percoll. F4/80 + macrophages were obtained by positive selection using magnetic MicroBeads. (**a**) qRT-PCR analysis was performed to characterize the phenotypes of macrophages. (**b**) The ratio of M2/M1 gene expression was calculated. Data were expressed as mean ± SEM. *p < 0.05. (**c**) The protein expression of iNOS (M1 marker) and CD206 (M2 marker) was analysed by immunofluorescent staining in control and fibrotic livers with or without acute insult.

We further probed why macrophages take on dichotomous roles in cases of homeostasis and hepatic fibrosis. As mentioned in the introduction, macrophages integrate various cues in the tissue microenvironment and shape their functional activation states accordingly. In other words, the liver microenvironment seems to be a decisive factor that determines the functional phenotype of macrophages^16, 18, 27^. We thereby presumed that the dichotomous roles of macrophages in these two cases are ascribed, to a great extent, to the discrepancy in their activation phenotypes. To characterize the macrophage phenotype under diverse circumstances, we isolated macrophages from the livers of control and fibrotic mice with or without acute insult, and subjected these cells to quantitative gene expression analysis by real-time PCR. As shown in Fig. 4a, M1 markers, including iNOS, CD86 and TNF-α, were significantly increased during acute injury, which was not the case in the fibrotic liver, even under acute challenge. On the other hand, M2 markers including arginase-1 (ARG-1) and CD206 were markedly up-regulated in the fibrotic liver. Nevertheless, these M2 signatures were unchanged or slightly increased in macrophages from the acutely injured liver. Of note, a high M2/M1 ratio was observed in the macrophages from the fibrotic livers when normalized to the expression of M1 genes (Fig. 4b). Thus, the balance between M1 and M2 signatures of macrophages was skewed into M2-preponderant activation in the context of liver fibrosis. To confirm this finding, we then carried out an immunofluorescence analysis of macrophage phenotype. In keeping with gene expression, acute insult triggered remarkable induction of the M1 marker iNOS (Green), whereas the M2 marker CD206 (Red) was moderately increased. By contrast, the fibrotic liver showed weaker expression of iNOS but stronger expression of CD206 (Fig. 4c). These data provide strong evidence that macrophages in the context of hepatic fibrosis exhibit M2-preponderant polarization. Notably, F4/80+ and CD206+ staining was co-localized within fibrotic septa as demonstrated by SMA+ staining (Supplementary Fig. 4). Furthermore, macrophages in the fibrotic liver subjected to Clo treatment and acute insult manifested stronger iNOS expression; conversely, control mice subjected to the same treatment displayed stronger CD206 expression (Supplementary Fig. 5). This result coincides with the aggravated hepatic damage in the former and alleviated liver injury in the latter. Thus, M2-preponderant macrophages might play a pivotal role in injury resistance in the fibrosis setting.

### M2-like macrophages confer hepatoprotection against acute challenge

**Figure 5:**
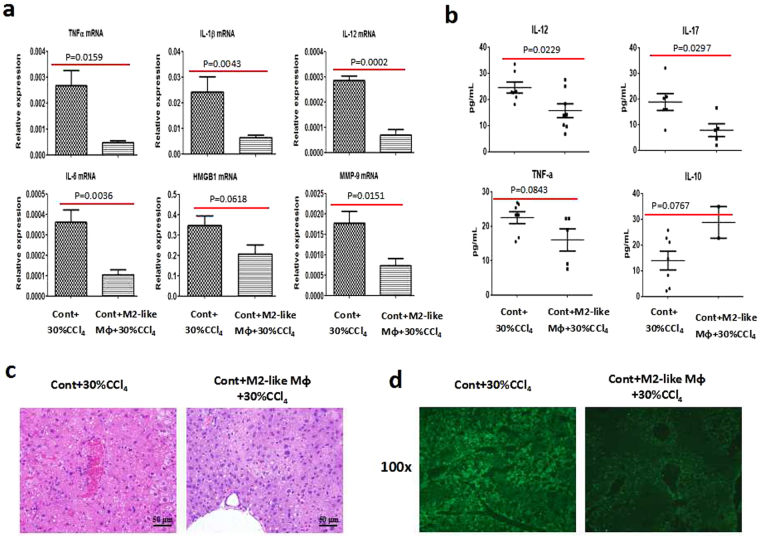
Adoptive transfer of M2-preponderant macrophages confers resistance to lethal insult. Macrophages isolated from the livers of fibrotic mice were adoptively transferred into control mice by tail vein. Twenty hours later, a lethal dose of CCl_4_ was given for an additional 24 hours. (**a** and **b**) The gene and protein levels of injury mediators were assessed by qRT-PCR and Luminex Assay, respectively. Data were expressed as mean ± SEM. Liver injury was visualized by H&E staining (**c**) and TUNEL assay (**d**).

To further confirm that the hepatoprotection occurring in the setting of hepatic fibrosis is conferred by M2-like macrophages, we performed a macrophage transfer experiment. M2-like macrophages isolated from the fibrotic liver were labelled with CFSE and then adoptively transferred into control mice by tail vein injection (~1 × 10^6^ cells/mouse). Twenty hours later, the efficiency of the adoptive transfer was verified by FACS analysis for CFSE-positive cells. The frequency of the CD11b+CFSE+/CD11b+ subset was 71.4%, showing that the majority of transferred macrophages were localized in the liver (Supplementary Fig. 6a). Then, the recipient mice were challenged with a dose of CCl_4_ (30%) for an additional 24 h. The hepatic damage was compared between mice with or without M2-like macrophage transfer. According to our data, adoptive transfer of M2-like macrophages led to a significant reduction in the mRNA levels of injury mediators, including TNF-α, IL-1β, IL-6, IL-12, HMGB1 and MMP-9 (Fig. 5a). Moreover, pro-inflammatory cytokines, including IL-12, IL-17 and TNF-α, decreased significantly or showed a decreased trend at the protein level in M2-like macrophage-transferred mice upon insult, as compared with acutely injured mice. Instead, the anti-inflammatory cytokine IL-10 showed an increased trend after M2-like macrophage transfer (Fig. 5b). Hepatoprotection was also identified by H&E staining and apoptosis detection. Mice that received M2-like macrophages manifested clearly alleviated hepatic pathological damage (Fig. 5c) and significantly reduced TUNEL-positive cells when exposed to acute insult (Fig. 5d). Moreover, control mice receiving M2-like macrophages exhibited stronger expression of CD206 upon insult, as opposed to stronger expression of iNOS in the acutely injured liver (Supplementary Fig. 6b). Collectively, the hepatoprotection against acute insult in the fibrosis setting is conferred by M2-like macrophages.

### M2-like macrophages protect hepatocytes against apoptosis *in vitro*

**Figure 6:**
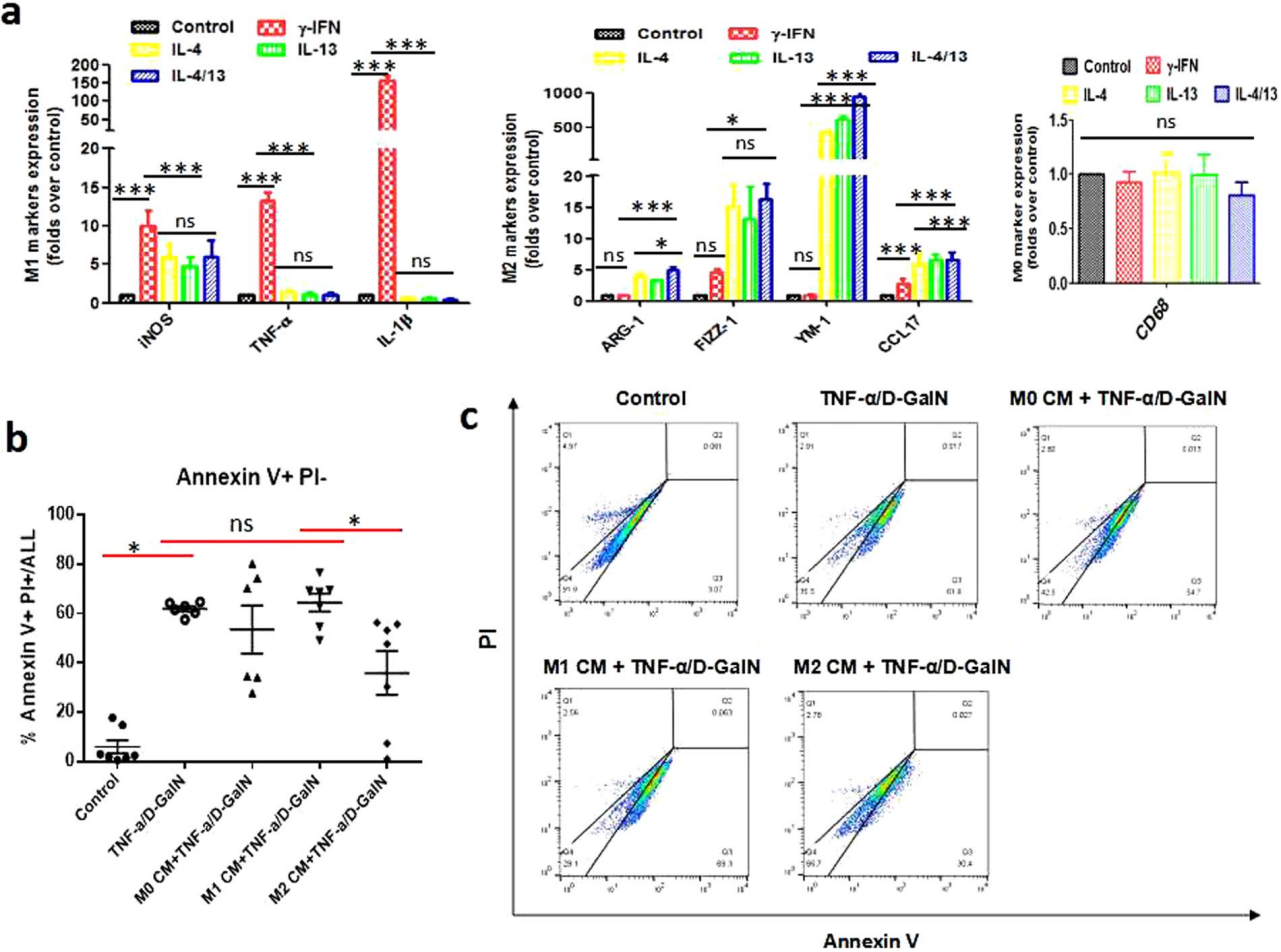
M2-polarized macrophages confer apoptosis resistance to hepatocytes (*in vitro*). M0 macrophages isolated from control mice were polarized *in vitro* by IFN-γ for M1 activation or IL-4/IL-13 for M2 activation. Then, conditioned media (CM) from M0, M1, and M2 macrophages were applied to primary hepatocytes for 6 hours, followed by apoptosis induction for another 12 hours. (**a**) The mRNA levels of M1 markers (iNOS, TNF-α, IL-1β), M2 markers (ARG-1, FIZZ-1, YM-1, CCL17), and a macrophage marker (CD68) were measured by qRT-PCR analysis. *p < 0.05, **p < 0.01, ***p < 0.001. (**b** and **c**) Hepatocyte apoptosis induced by TNF-α/D-GalN was quantitatively analysed by Annexin V-FITC/PI double-labelled flow cytometry. *p < 0.05. Data were expressed as mean ± SEM.

Finally, we dissected the mechanism by which M2-like macrophages confer hepatoprotection through an *in vitro* experiment. A significant induction of M1 signatures, including iNOS, TNF-α and IL-1β, was detected in macrophages treated with IFN-γ. On the other hand, M2 signatures, especially YM-1, were substantially induced by IL-4 and/or IL-13 (Fig. 6a). Thus, *in vitro* polarization of primary mouse macrophages was induced successfully. Then, conditioned media (CM) from control (M0 macrophages), polarized M1 and M2 macrophages were applied to primary hepatocytes followed by apoptosis induction. Hepatocyte apoptosis was quantitatively analysed by Annexin V-FITC/PI double-labelled flow cytometry. As expected, TNF-α/D-GalN triggered massive apoptosis of hepatocytes, as shown by the high frequency of Annexin V+/PI− apoptotic cells. Exposure of primary hepatocytes to either M0 CM or M1 CM had no significant effect on cell apoptosis. Nevertheless, the frequency of apoptosis was substantially reduced (from 61.95 ± 1.10 to 35.97 ± 8.88 for Annexin V+/PI− staining) in hepatocytes with M2 CM pretreatment (Fig. 6b and c). Therefore, M2-like macrophages confer apoptosis resistance to hepatocytes.

## Discussion

Hepatoprotection in the context of liver fibrosis is an intriguing issue that remains to be fully elucidated. In the present work, we dissected this issue from a novel viewpoint, i.e., macrophages and their M1/M2 activation. Herein, we provide powerful evidence that M2-like macrophages in the fibrotic liver exert beneficial hepatoprotection against acute insult by conferring apoptosis resistance to hepatocytes. To the best of our knowledge, this is the first study linking macrophage M1/M2 activation with injury resistance in the setting of liver fibrosis.

Fibrosis is widely acknowledged as a damaging process with potential progression to cirrhosis and further sequelae. Remarkably, recent studies have reported the hepatoprotective effects occurring in the context of liver fibrosis. In a mouse model of partial bile duct ligation (PBDL), the pre-injured lobes (fibrotic) exhibit better tolerance to TNF-α- and Fas-induced hepatocyte apoptosis compared with non-ligated lobes^6^. Another outstanding study showed that thioacetamide (TAA)-induced liver fibrosis is less vulnerable to a wide variety of injurious stimuli^8^. In particular, a robust and self-limiting fibrotic and regenerative response is also observed in the context of acute liver injury^28^. In the present study, we characterized injury resistance using a liver fibrosis model induced by CCl_4_ injection. Similar hepatoprotection against acute insult, especially lethal injury inflicted by D-GalN/LPS and APAP, was found in our work. The injury resistance occurs in the setting of liver fibrosis and recedes along with fibrosis resolution (Bai *et al*., unpublished data), favouring the theory that hepatic fibrosis is bound up with hepatoprotection. Given that injury resistance is broad-spectrum, we speculated that innate immunity may be the major contributor to injury resistance in the fibrosis setting. As expected, fibrotic mice upon challenge showed the absence of pro-inflammatory immune responses and the suppression of MMPs compared with the acutely injured liver. HMGB1 occupies a central role in mediating cell death and innate immunity^24, 29, 30^. To function as a DAMP molecule, HMGB1 is passively released from necrotic cells or actively secreted from inflammatory cells after hyperacetylation^31, 32^. In our work, massive translocation of HMGB1 arising in acutely injured livers was extremely inhibited in the fibrotic livers, even under insult. Collectively, injury resistance in the setting of hepatic fibrosis is accompanied by innate immune tolerance. Of note, resistance to toxins in the fibrotic liver could be manifested by decreased drug metabolism. To sort out such a possibility, we measured the hepatic levels of Cyp2E1. As shown in Supplementary Fig. 1c, there was no difference in the mRNA levels of Cyp2E1 between acute and chronic liver injury, which excludes its role in injury resistance.

The liver is a central immunological organ which maintains immune suppression and hepatic tolerance in homeostasis. Macrophages play a principal role in tolerance induction and pathogen clearance^15, 33^. In PBDL model as mentioned above^7^, KC depletion by alendronate liposomes increased hepatocyte damage and the susceptibility to TNF-α-induced hepatocyte apoptosis in ligated lobes. However, KC depletion did not affect the hepatic injury in the non-ligated lobes. Interestingly, in our macrophage depletion experiments, divergent roles were observed: depletion of macrophages in the control mice ameliorated liver injury upon acute challenge, which was accompanied by the down-regulation of pro-inflammatory mediators and significant reduction of apoptotic cells; conversely, exhaustion of macrophages in the fibrotic mice exacerbated liver injury upon insult, which was consistent with the breakdown of immune tolerance, the restoration of pro-inflammatory immune responses, as well as massive apoptosis. The difference in the responsiveness of macrophage-depleted control mice or non-ligated lobes to injurious stimulus might be explained by the disparities in terms of animal model, reagents for macrophage depletion and acute challenge, and treatment time.

Why do macrophages in control and fibrotic mice exhibit contradictory roles? We speculated that it could be attributed, at least in part, to the heterogeneity of macrophages. As mentioned in the introduction, M1 macrophages arise in inflammatory settings dominated by Toll-like receptor (TLR) and interferon signaling, and exhibit potent pro-inflammatory and antimicrobial activities; M2 macrophages are found in TH2-dominated settings such as helminths infection, and display the anti-inflammatory and tissue-reparative activities^17^. Although the use of terms M1 and M2 remains controversial because of the lack of tightly defined criteria to score phenotypes, M1/M2 paradigm provides an outline with which to distinguish the states and functions of macrophages. Importantly, the activation states of macrophages are not fixed, instead, they integrate multiple signals from tissue microenvironment and shape their phenotype accordingly. That is to say, macrophages *in vivo* adopt a mixed phenotype between M1- and M2-type macrophages. In this regard, the M1/M2 balance seems to be a decisive factor for macrophage function^17, 18, 34^. In our work, macrophages from the fibrotic liver exhibit high M2/M1 ratio at the gene (higher expression of M2 markers when normalized to the expression of M1 genes by PCR assay) and protein (stronger expression of M2 marker CD206 by IF staining) levels. Thus, the balance between M1 and M2 activation is probably skewed toward an M2-preponderant phenotype in fibrosis setting. This finding is agreed with the latest study by Bility and coworkers. They characterized macrophage polarization in chronic HCV-induced liver inflammation and fibrosis, and showed that M2 macrophage activation was associated with liver fibrosis in humanized mice and patients^35^. However, a previous report demonstrated that M2-like macrophages in the liver are associated with accelerated liver fibrosis and necrosis in patients with acute HBV-induced liver failure^36^. This conclusion seems to be inconsistent with our data. We speculate that this divergence is likely ascribed to HBV, which promotes M2 macrophage polarization in both M1 and M2 macrophages in humans.

We further confirmed the hepatoprotection of M2-like macrophages through macrophage transfer experiment. Transfusion of M2-like macrophages isolated from the fibrotic liver conferred the control mice more resistance to acute challenge, as evidenced by decreased expressions of pro-inflammatory mediators (at the gene and protein levels), reduced apoptotic cells and better-preserved liver architecture. Thus, M2-like macrophages did protect mice against acute challenge. How do M2-like macrophages exert the hepatoprotection? Wan and coworkers investigated the impact of alternatively activated M2 KC on alcohol- and high fat-induced liver injury. They unraveled a novel mechanism by which M2 KCs protect against alcoholic liver disease (ALD) and non-alcoholic fatty liver disease (NAFLD), namely, M2 KCs promote M1 KCs apoptosis^34^. They also reported M2-polarized macrophages trigger hepatocyte senescence *in vivo* and *in vitro*, which is associated with resistance to ethanol-induced apoptosis^37^. In this regard, we analysed the effects exerted by M2-like macrophages on hepatocyte apoptosis. TUNEL-positive staining was noticeably reduced in both the fibrotic liver upon insult and M2-like macrophage-transferred liver compared with the acutely injured liver, supporting the intimate association between M2-like macrophages and apoptosis resistance. Importantly, our *in vitro* data showed that M2-like macrophages protect hepatocytes against apoptosis, as indicated by a significant reduction in Annexin V+/PI− staining in primary hepatocytes treated with M2 CM. Therefore, the hepatoprotection in the setting of liver fibrosis can be mediated by M2-like macrophages through inhibiting hepatocyte apoptosis.

There are several limitations in our work: (1) Current findings are preliminary, and further studies should be performed to address the molecular basis of the hepatoprotection conferred by M2-like macrophages. Enhanced understanding of complex factors that govern tissue macrophage plasticity may allow manipulation of macrophage function to shorten the duration of inflammation and expedite tissue repair^38^. (2) Macrophage polarization is a complex and dynamic process. Our work only takes a “snapshot” at some point of injury resistance in the setting of liver fibrosis. Therefore, the time-dependence of hepatoprotection and injury tolerance should be analysed in future work.

In summary, we have shown M2-like macrophage originating from liver fibrosis is a pivotal contributor for the beneficial hepatoprotection against lethal insults. At present, dysregulation of the M1/M2 phenotypic balance is emerging as a central mechanism governing the pathogenesis of acute and chronic inflammatory diseases^34^. Early re-orientation of hepatic macrophage function towards a ‘pro-resolving’ phenotype represents a promising and attractive therapy strategy^38^.

## Methods

### Animals

Male BALB/c mice (6–8 weeks old; 20–25 g) were purchased from Laboratory Animal Center, Academy of Military Medical Sciences, Beijing, China. Mice were housed in a specific pathogen free (SPF) environment under controlled conditions (22 °C–24 °C, 12-hour light/dark cycle). Animals were fed standard laboratory chow with free access to water. All animal care and experimental procedures performed in this study were in accordance with the Guide for the Care and Use of Laboratory Animals and were approved by Institutional Animal Care and Use Committee of Beijing YouAn Hospital affiliated to Capital Medical University.

### Animal treatment

Mice were treated as follows: (1) Control: BALB/c mice were injected intraperitoneally with mineral oil as a control. The administration of mineral oil had no marked effect on liver function, even after 6 weeks (Supplementary Fig. 1a). (2) Induction of hepatic fibrosis: BALB/c mice were injected intraperitoneally with CCl_4_ in mineral oil, twice a week for 6 weeks. The initial dose of CCl_4_ was 0.2 μl/g (2%), and the doses were then increased gradually up to 3 μl/g (30%). The development of liver fibrosis was identified by Masson staining (Supplementary Fig. 1b). (3) Acute challenge: Control and fibrotic mice were challenged through intraperitoneal injection with a lethal dose of hepatic toxins: D-GalN (1 mg/g)/LPS (50 ng/g), APAP (1 mg/g), or CCl_4_ (3 μl/g). Sera and liver tissues were harvested 24 hours after acute challenge for analysis. A portion of the liver was fixed in 10% neutral-buffered formalin for histological analysis and immunostaining. The remaining liver was cut into pieces and snap-frozen for homogenization to extract total liver RNA and protein.

### Evaluation of liver injury

Serum ALT and AST levels were measured using a multiparametric analyser (AU 5400, Olympus, Japan) according to an automated procedure.

Liver tissues fixed in 10% formalin were embedded in paraffin, sectioned, and stained with haematoxylin-eosin (H&E) and Masson’s trichrome for light microscopy. Histological severity of hepatic damage was assessed and scored blindly by experienced pathologists according to Modified Histological Activity Index^39^.

### Terminal deoxynucleotidyl transferase dUTP nick end labeling (TUNEL) assay

TUNEL assay was performed on frozen liver sections using an *in situ* cell death detection kit (fluorescein) (Roche Diagnostics GmbH, Mannheim, Germany).

### SYBR Green quantitative reverse-transcriptase polymerase chain reaction (qRT-PCR)

Total RNA was extracted from frozen liver tissues using TRIzol reagent (Invitrogen, Carlsbad, CA, USA). cDNA was synthesized from 2 μg of RNA using random primers and an AMV retrotranscriptase system (TakaRa, Dalian, Liaoning, China). SYBR Green RT-PCR was carried out using the ABI StepOne Plus Real-Time PCR System (Applied Biosystems, Foster City, CA, USA). All reactions were performed in triplicate. In a final reaction volume of 20 μl, the following were added: 1′ SYBR Green (TakaRa, Dalian, Liaoning, China), cDNA, 0.5 mM each primer, and ROX. The conditions of the reaction were as follows: 50 °C (2 min), 95 °C (5 min), followed by 40 cycles of 95 °C (15 s) and 60 °C (30 s). The primers were designed by Primer version 3.0 and are listed in Table 1. The relative expression of target genes was calculated and normalized to the expression of GAPDH, a housekeeping gene.

**Table 1:** Primer sequences for real-time PCR.

Genes	Sense	Anti-sense
GAPDH	5′-AACTTTGGCATTGTGGAAGG-3′	5′-ACACATTGGGGGTAGGAACA-3′
MMP-9	5′-CGTGTCTGGAGATTCGACTTGA-3′	5′-TGGAAGATGTCGTGTGAG-3′
MMP-13	5′-GGCCAGAACTTCCCAACCAT-3′	5′-GAACCGCAGCGCTCAGTCTCT-3′
IL-1β	5′-GCCCATCCTCTGTGACTCAT-3′	5′-AGGCCACAGGTATTTTGT-3′
IL-6	5′-AGTTGCCTTCTTGGGACTGA-3′	5′-TCCACGATTTCCCAGAGAAC-3′
IL-12p40	5′-CAGCTTCTTCATCAGGGACAT-3'	5′-CTTGAGGGAGAAGTAGGAATGG-3'
TNF-α	5′-GCCTCTTCTCATTCCTGCTTGT-3'	5′-TTGAGATCCATGCCGTTG-3'
CD206	5′-ATGCCAAGTGGGAAAATCTG-3′	5′-TGTAGCAGTGGCCTGCATAG-3′
iNOS	5′-CGGAGCCTTTAGACCTCAACA-3′	5′-CCCTCGAAGGTGAGCTGAAC-3′
ARG-1	5′-CTGGCAGTTGGAAGCATCTCT-3′	5′-GTGAGCATCCACCCAAATGAC-3′
FIZZ1	5′-CCTCCACTGTAACGAAGACTCTC-3′	5′-GCAAAGCCACAAGCACACC-3′
YM-1	5′-ATCTATGCCTTTGCTGGAATGC-3′	5′-TGAATGAATATCTGACGGTTCTGAG-3′
CCL17	5′-TGCTTCTGGGGACTTTTCTG-3′	5′-TGGCCTTCTTCACATGTTTG-3′
CD68	5′-TTCTGCTGTGGAAATGCAAG-3′	5′-AGAGGGGCTGGTAGGTTGAT-3′
CD86	5′-CACGAGCTTTGACAGGAACA-3′	5′-TTAGGTTTCGGGTGACCTTG-3′
HMGB1	5′-ATGGGCAAAGGAGATCCTA-3′	5′-ATTCATCATCATCATCTTCT-3′

### Gelatine zymography

Frozen liver tissues (100 mg) were homogenized in 1.0 ml ice-cold NT buffer (100 mM NaCl, 50 mM Tris-HCl, pH 7.5) containing 1% Triton X-100. The homogenates were centrifuged at 12,000 rpm for 20 min at 4 °C. To enrich gelatinases for zymography analysis, samples with equal amounts of total protein were concentrated with gelatine Sepharose^TM^ 4B (GE Healthcare Bio-Sciences AB, Uppsala, SE) according to the manufacturer’s instructions. Purified proteins were separated by 10% sodium dodecyl sulfate-polyacrylamide gel electrophoresis (SDS-PAGE) containing 1 mg/ml of bovine skin gelatine (Sigma, St Louis, MO, USA). Then, the gel was re-natured in 2.5% Triton X-100 for 30 min, followed by incubation in activity buffer (50 mM Tris, pH 7.5, 200 mM NaCl, 5 mM CaCl_2_) at 37 °C for 16–24 hours. The gel was visualized by 0.5% Coomassie-R250 staining followed by destaining in 40% methanol and 10% acetic acid. MMP-9 and MMP-2 gelatinolytic activity was detected as destained bands against a dark blue background of stained gelatine. The active form of MMP-9 was designated as a 92 kDa band.

### Immunofluorescence and immunohistochemical analysis

Immunostaining was performed on frozen liver sections or paraffin-embedded sections, as previously described^40^. Liver sections were stained with the following primary antibodies: FITC mouse anti-iNOS/NOS Type II (610330; Clone: 6/iNOS/NOS Type II; 1:400; BD Transduction Laboratories™, San Jose, CA, USA), PE anti-mouse CD206 (MMR) (141705; Clone:C068C2; 1:400; BioLegend Inc., San Diego, CA, USA), anti-mouse F4/80 antigen PerCP-Cyanine5.5 (45–4801; Clone: BM8; 1:300; eBioscience, San Diego, CA, USA), anti-actin, α-smooth muscle-FITC antibody (F3777; Clone: 1A4; 1:300; Sigma, St Louis, MO, USA), and anti-mouse CD206:Alexa Fluor®488 (MCA2235A488T; Clone: MR5D3; 1:400; AbD Serotec, Raleigh, NC, USA). A Nikon Inverted Fluorescence Microscope ECLIPSE Ti and NIS-Elements F 3.0 Software (Nikon Corporation, Tokyo, Japan) as well as an Olympus Bx51 microscope (Olympus America, Melville, NY, USA) and CellSens standard 1.4.1 software were applied for image capture in immunohistochemical analysis and immunofluorescence, respectively.

### Luminex Assay

Serum samples were analysed for cytokine/chemokine concentrations using the Mouse Cytokine/Chemokine Magnetic Bead Panel (MCYTOMAG-70K, EMD Millipore Corporation, St. Charles, Missouri, USA) Luminex Assay according to the manufacturer’s instructions.

### Isolation and purification of primary hepatocytes and macrophages

Hepatocytes and hepatic nonparenchymal cells (NPC) were isolated from mice by pronase (Roche Diagnostics GmbH, Mannheim, Germany) and collagenase (Sigma-Aldrich, St. Louis, MO, USA) digestion followed by differential centrifugation using the previously reported method with some modifications^41^. Briefly, *in situ* perfusion was applied through the portal vein and superior vena cava with 0.9% NaCl followed by DMEM/F12 (Gibco, Grand Island, NY, USA) containing 0.5% Pronase and DMEM/F12 containing 0.04% type IV collagenase. Then, the liver was harvested, excised, and digested with DMEM/F12 containing 10 μg/ml DNase (Sigma-Aldrich, St. Louis, MO, USA). Digested livers were passed through a 70-μm cell strainer (BD Falcon). The filtrate was centrifuged, the pellets were harvested and washed (primary hepatocytes), and the supernatant was centrifuged to acquire macrophages. The resultant pellets were re-suspended in DMEM (HyClone, Logan, Utah, USA), overlaid onto Percoll (Sigma, Sigma-Aldrich, St. Louis, MO, USA) gradients (40% to 70%), and centrifuged at 1,100 × g for 20 min. Liver NPC suspensions were further overlaid onto a Percoll gradient (25% to 50%) and centrifuged at 1,800 × g for 30 min. The interface was harvested and stained with biotin-conjugated anti-F4/80 (eBiosciences, San Diego, CA, USA). F4/80+ macrophages were further purified by streptavidin-conjugated magnetic beads (Miltenyi Biotec, Auburn, CA, USA) according to the manufacturer’s protocols. This procedure routinely yielded (2.5–3) × 10^5^ cells/liver.

### Depletion of macrophages followed by acute insult

Liposome-encapsulated clodronate (Clo) (FormuMax Scientific Inc., Palo Alto, USA) was used to deplete macrophages. A single injection of 0.15 ml Clo through the tail vein was able to deplete F4/80 + cells in the liver to maximum levels at 48 h (Supplementary Fig. 3b). Clo did not lead to obvious liver damage, as assessed by serum ALT levels (Supplementary Fig. 3c). The mice injected with the same dose of phosphate-buffered saline (PBS)-encapsulated liposome (PBS-lip) (FormuMax Scientific Inc., Palo Alto, USA) were used as controls.

To probe the functions of macrophages in injury resistance and immune tolerance, control and fibrotic mice were subjected to Clo treatment for 48 h, then the resultant mice were challenged by 30%CCl_4_. Serum and liver tissues were harvested for analysis at 24 h after acute insult.

### *In vitro* polarization of macrophages and conditioned medium (CM) experiments

Macrophages isolated from control mice (non-polarized M0 macrophages) were cultured in DMEM medium containing 10% FBS. The cells were stimulated with IFN-γ (100 U/ml, PeproTech, Rocky Hill, USA) for 36–48 h for M1 induction or IL-4/IL-13 (10 ng/ml each, PeproTech) for M2 induction. The phenotype of the subsets was identified through qRT-PCR analysis for gene signatures of representative markers. Then, CMs from M0 macrophages (M0 CM), M1 macrophages (M1 CM) or M2 macrophages (M2 CM) were collected and centrifuged to remove cell debris. Afterwards, CMs were incubated with primary hepatocytes isolated from normal mice for 6 hours and subjected to apoptosis induction.

### The induction and detection of hepatocyte apoptosis *in vitro*

Hepatocyte apoptosis was induced by TNF-α (50 μg/ml)/D-GalN (100 mg/ml) for 12 hours. To evaluate cell apoptosis, primary hepatocytes were stained with PI/Annexin V (FITC Annexin V Apoptosis Detection Kit II, BD Pharmingen, San Jose, CA, USA), and apoptosis was detected by FACS according to the manufacturer’s instruction. The data were analysed by FlowJo v10 software (TreeStar Inc., Ashalnd, OR, USA).

### Statistical analysis

The results were expressed as the means ± standard error of the mean. Group comparisons were performed using Student’s *t* test, Mann-Whitney U test, or one-way ANOVA, as appropriate. The survival rates were calculated using the Kaplan-Meier method, and survival curves were compared using the log-rank test. Statistics and graphs were generated using Prism 5.0 software (GraphPad Software Inc., San Diego, CA, USA). p < 0.05 was considered statistically significant.

## Electronic supplementary material

Supplementary information
