# Supplementary material for: M2-like macrophages in the fibrotic liver protect mice against lethal insults through conferring apoptosis resistance to hepatocytes
Source: Sci Rep. 2017 Sep 5;7:10518. doi: 10.1038/s41598-017-11303-z (PMC5585332; doi:10.1038/s41598-017-11303-z)
Supplement: Supplementary file 1 — Supplementary information [file 41598_2017_11303_MOESM1_ESM.pdf]

M2-like macrophages in the fibrotic liver protect mice against lethal insults through conferring apoptosis resistance to hepatocytes

Li Bai[1], Xin Liu[1], Qingfen Zheng[1], Ming Kong[1], Xiaohui Zhang[1], Richard Hu[2], Jinli Lou[1], Feng Ren[3], Yu Chen[1], Sujun Zheng[1], Shuang Liu[1], Yuan-Ping Han[4,*], Zhongping Duan[1,*], and Stephen J. Pandol[5]

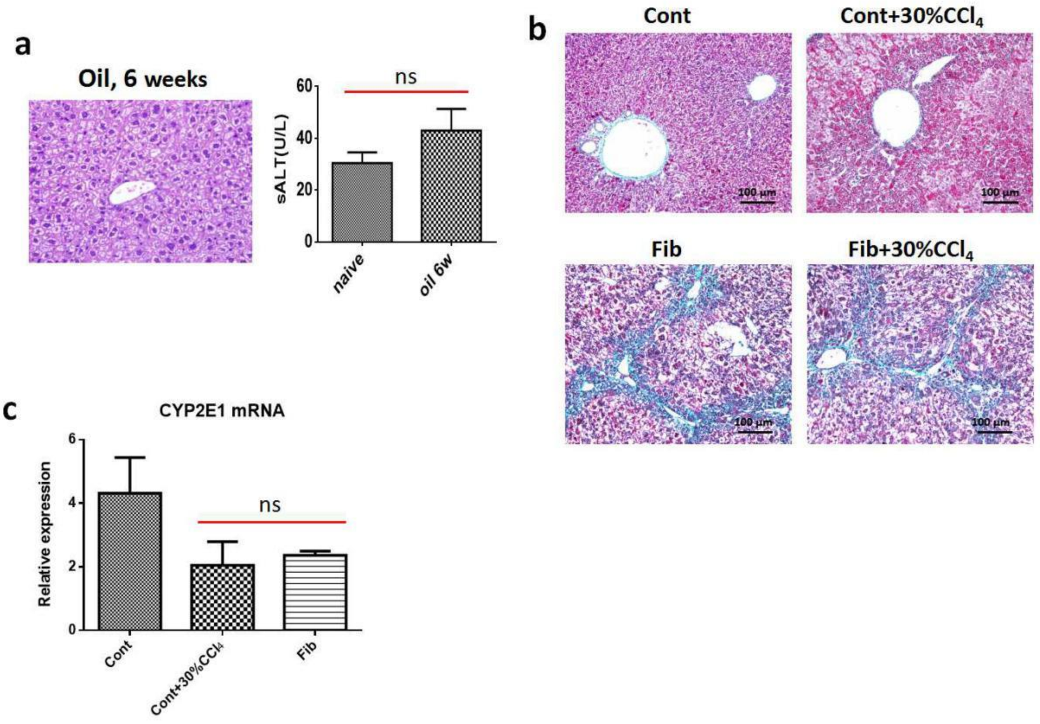

Supplementary Figure 1. Control and fibrotic (CCl_4, 6 weeks) BalBc mice were challenged with CCl_4 (3 μl/g). (a) The injection of mineral oil did not lead to marked liver injury, even for 6 weeks, which was shown by histologic pathology and serum ALT levels. (b) Fibrosis was successfully induced according to Masson staining of representative liver tissues. (c) The expression of hepatic Cyp2E1 was similar in acute and chronic liver injury.

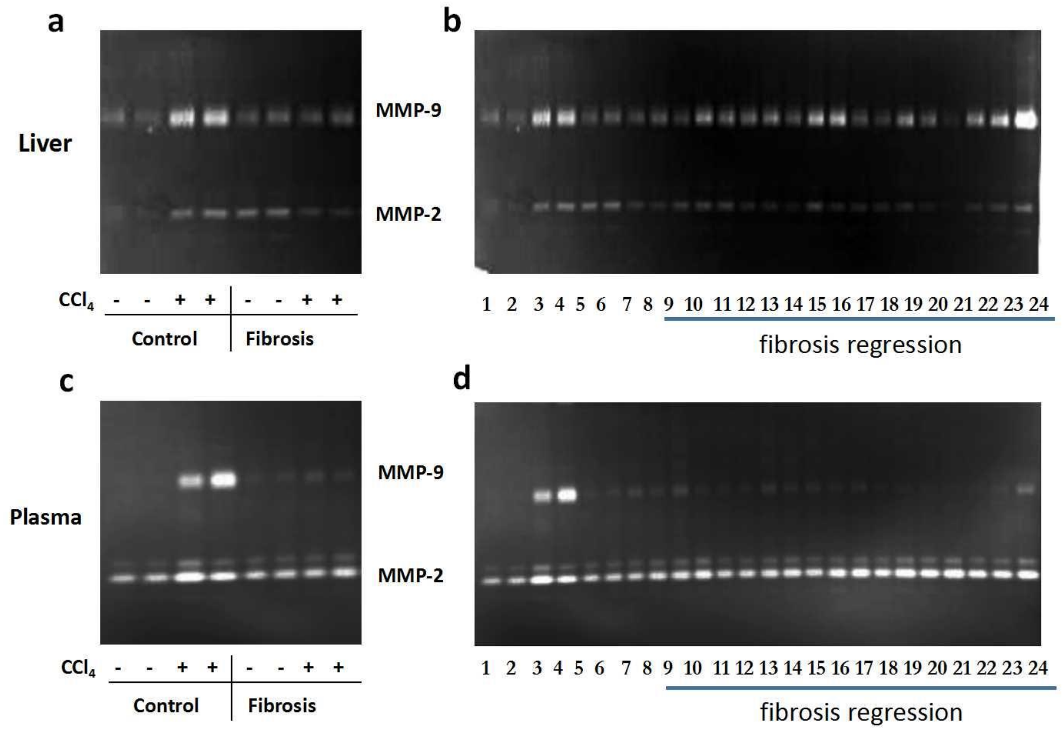

Supplementary Figure 2. The MMP-9 activities in the liver tissues and plasma were detected by zymography. Figure 2a (liver) and 2c (plasma) were derived from Figure 2b (liver) and 2d (plasma).

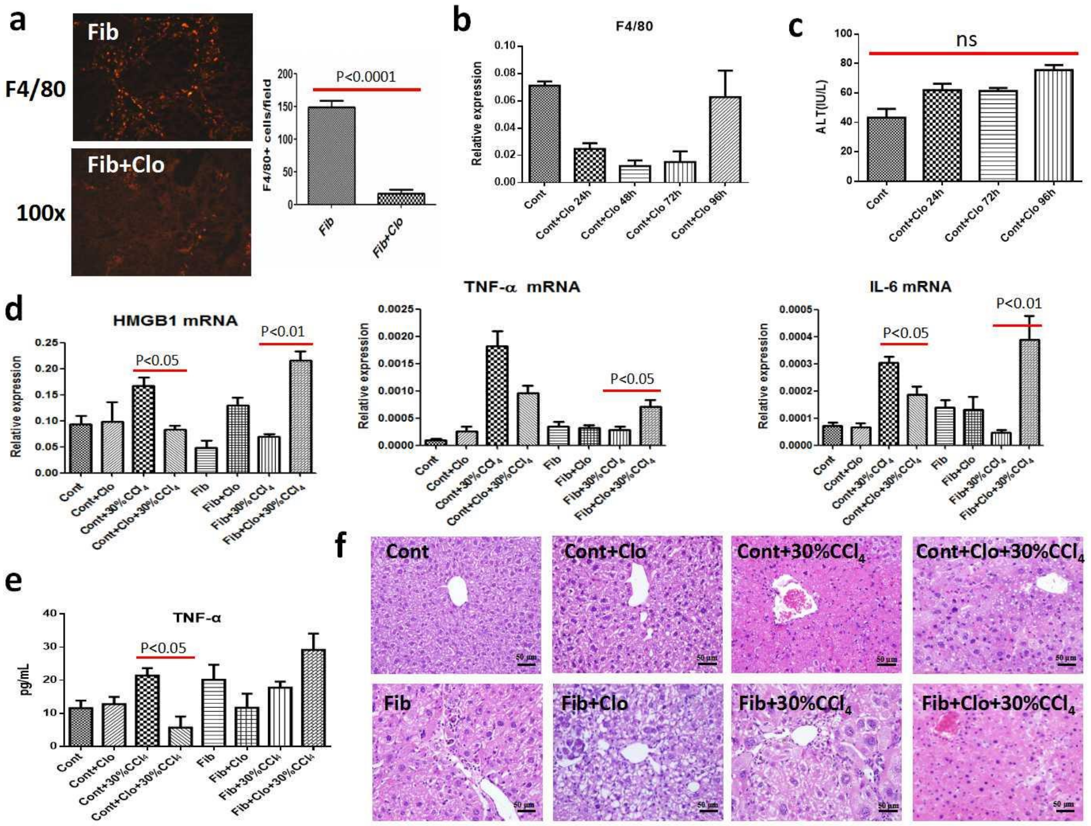

Supplementary Figure 3. Macrophages in the control and fibrotic mice were depleted with liposome-clodronate (Clo), and the resultant mice were then challenged with a lethal dose of CCl_4. Sera and liver tissues were harvested 24 hours after toxin challenge. (a) The depletion of macrophages in the fibrotic liver was verified by F4/80 staining. (b) Time course experiment of macrophage depletion was monitored by RT-qPCR analysis for F480. (c) The administration of liposome-clodronate (Clo) did not cause significant liver damage as determined by serum ALT. (d and e) The mRNA and protein expressions of injury markers were detected by qRT-PCR and Luminex Assay, respectively. (f) H&E staining of representative liver tissues. Histological severity of liver injury was assessed and scored blindly by experienced pathologists.

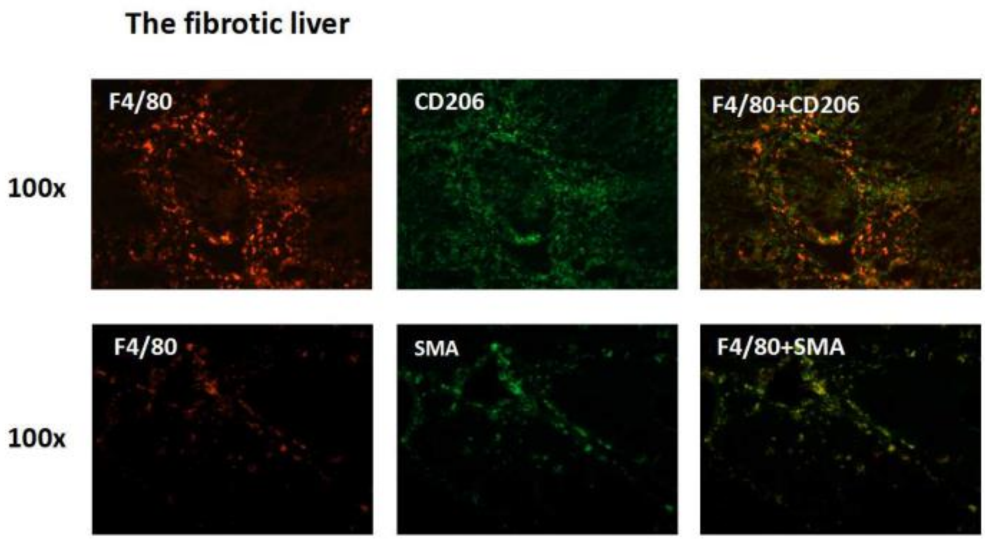

Supplementary Figure 4. F4/80, CD206, and smooth muscle actin (SMA) were co-localized in the fibrotic liver by immunofluorescence.

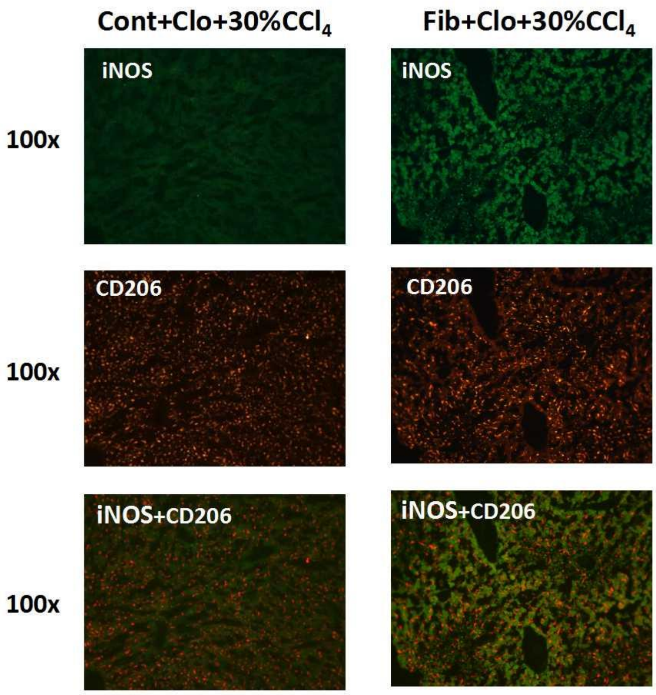

Supplementary Figure 5. Control and fibrotic mice were subjected to liposome-clodronate (Clo) treatment, and then challenged with acute insult (30%CCl_4). Liver tissues were harvested at 24 hours after challenge. The expression of M1 marker iNOS and M2 marker CD206 was detected by immunostaining.

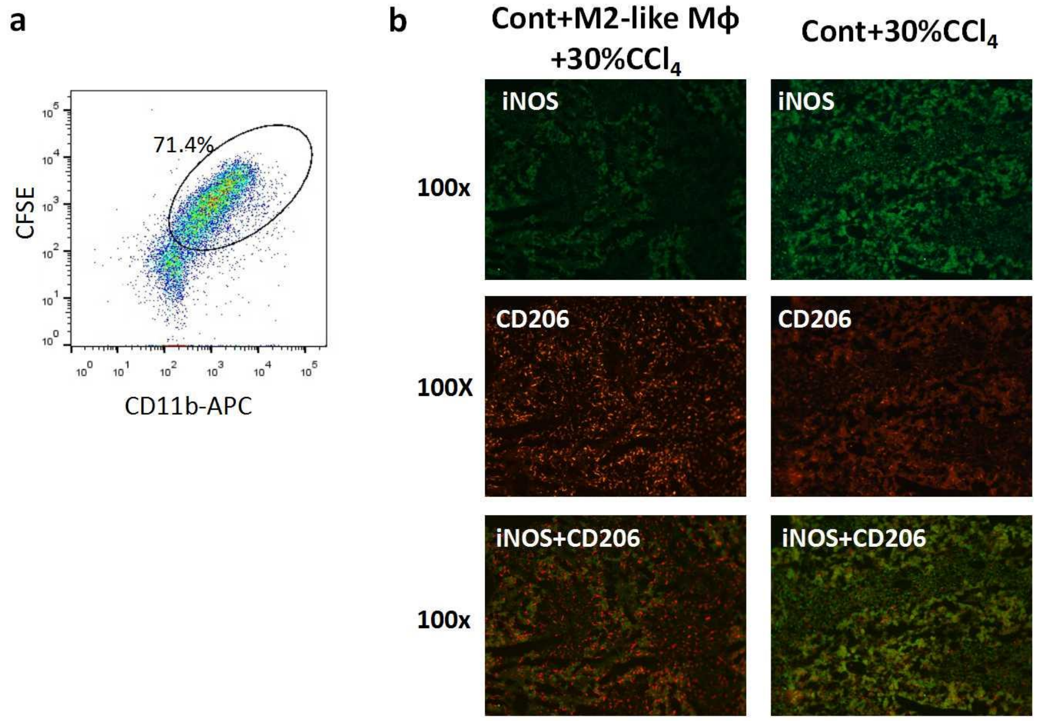

Supplementary Figure 6. (a) The macrophages isolated from the fibrotic liver were labeled with CFSE, then transferred into the control mice. Twenty-hours after transfer, macrophages were isolated and detected by FACS. (b) Macrophages isolated from the liver of fibrotic mice were adoptively transferred into the control mice by tail vein. Twenty hours later, a lethal dose of CCl_4 was given for additional 24 hours. The expression of M1 marker iNOS and M2 marker CD206 in the liver was detected by immunostaining.
